# Ortho­rhom­bic polymorph of (2*E*)-2-(2,3-dihydro-1*H*-inden-1-yl­idene)-2,3-di­hydro-1*H*-inden-1-one^1^

**DOI:** 10.1107/S1600536812035994

**Published:** 2012-08-23

**Authors:** Hairong Li, Frank R. Fronczek, Steven F. Watkins

**Affiliations:** a Department of Chemistry, Louisiana State University, Baton Rouge, LA 70803-1804, USA

## Abstract

The title compound, C_18_H_14_O, is polymorphic at 123 K. The ortho­rhom­bic form reported herein has two independent mol­ecules in the asymmetric unit, with mol­ecular volume 313.5 Å^3^. The previously reported triclinic (*P*-1) form [Raston & Scott (2000 ▶). *Green Chem.*, **2**, 49–52] has mol­ecular volume 309.6 Å^3^ at the same temperature. All three mol­ecules deviate significantly and systematically from the putative *C_s_* symmetry (δ_r.m.s._ = 0.0265, 0.0256, 0.0497 Å). Comparison of the two molecules in the orthorhombic polymorph shows that 16 of the 19 equivalent pairs of framework atoms have a mirror-image pattern of deviations (above/below plane), suggesting that the two are quasi-enanti­omorphs. The pattern of deviations in the triclinic form is nearly the same (13 of 19 atom pairs) as the ortho­rhom­bic form.

## Related literature

For the title compound co-crystallized with 2,4-di-*tert*-butyl­phenol, see: Turner *et al.* (2003 ▶; CSD refcode IQAZAB). For the Cambridge Structural Database (CSD), see: Allen (2002 ▶). For the determination of an absolute structure from Bijvoet pairs, see: Hooft *et al.* (2008 ▶). For the synthesis of the title compound, see: Bell & Spanswick (1966 ▶). For *cis*-*trans* isomerism in the title compound, see: Williams (1967 ▶).
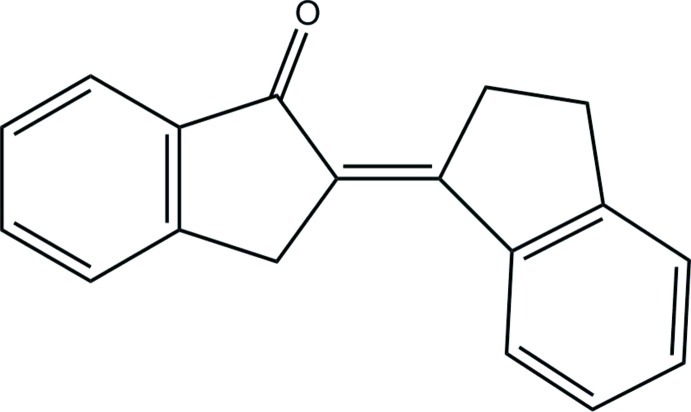


## Experimental

### 

#### Crystal data

C_18_H_14_O
*M* *_r_* = 246.29
Orthorhombic, 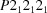

*a* = 5.291 (2) Å
*b* = 17.809 (5) Å
*c* = 26.622 (9) Å
*V* = 2508.5 (15) Å^3^
*Z* = 8
Mo *K*α radiation
μ = 0.08 mm^−1^
*T* = 123 K
0.35 × 0.12 × 0.05 mm

#### Data collection

Nonius KappaCCD diffractometer
Absorption correction: multi-scan *HKL* *SCALEPACK* (Otwinowski & Minor 1997 ▶) *T*_min_ = 0.973, *T*_max_ = 0.996
11743 measured reflections
4236 independent reflections
3145 reflections with *I* > 2σ(*I*)
*R*_int_ = 0.074

#### Refinement

*R*[*F* ^2^ > 2σ(*F* ^2^)] = 0.057
*wR*(*F* ^2^) = 0.110
*S* = 1.05
4236 reflections
344 parameters
H-atom parameters constrained
Δρ_max_ = 0.21 e Å^−3^
Δρ_min_ = −0.18 e Å^−3^
Absolute structure: Flack (1983 ▶), with 1665 Bijvoet pairs
Flack parameter: 0 (2)

### 

Data collection: *COLLECT* (Nonius, 2000 ▶); cell refinement: *DENZO* and *SCALEPACK* (Otwinowski & Minor, 1997 ▶); data reduction: *DENZO* and *SCALEPACK*; program(s) used to solve structure: *SIR2002* (Burla *et al.*, 2003 ▶); program(s) used to refine structure: *SHELXL97* (Sheldrick, 2008 ▶); molecular graphics: *ORTEP-3 for Windows* (Farrugia, 1997 ▶); software used to prepare material for publication: *IDEAL* (Gould *et al.*, 1988 ▶) and *WinGX* (Farrugia, 1999 ▶).

## Supplementary Material

Crystal structure: contains datablock(s) global, I. DOI: 10.1107/S1600536812035994/jj2149sup1.cif

Structure factors: contains datablock(s) I. DOI: 10.1107/S1600536812035994/jj2149Isup2.hkl

Supplementary material file. DOI: 10.1107/S1600536812035994/jj2149Isup3.cml

Additional supplementary materials:  crystallographic information; 3D view; checkCIF report

## Figures and Tables

**Fig. 1.**
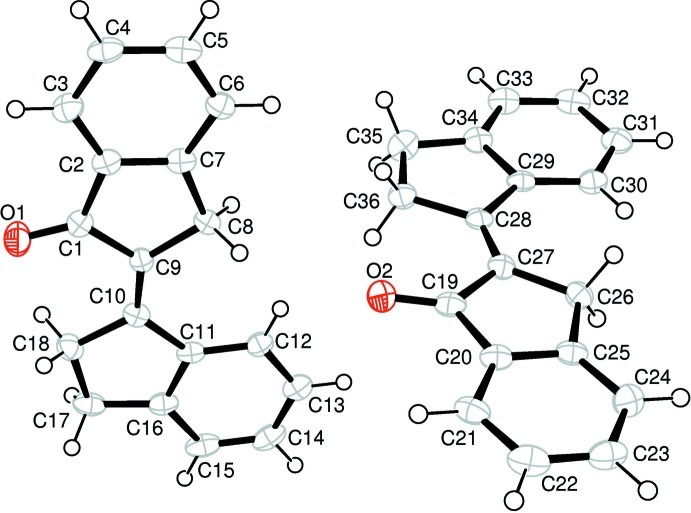
View of (I) (50% probability displacement ellipsoids)

## supplementary crystallographic information

### Comment

Four pairs (Ia, Ib) of independent molecules of the title compound occupy an orthorhombic unit cell at 123 K. Thirteen non-H framework atoms of Ia, and sixteen atoms of Ib, deviate significantly from their mean planes: δ_max_ = 0.053 (3) and δ_r.m.s._ = 0.027 Å, δ_max_ = 0.052 (3) and δ_r.m.s._ = 0.026 Å respectively. A least-squares fit of chemically equivalent pairs of non-H atomic positions (*IDEAL*, Gould *et al.*, 1988) shows an average mis-match δ_r.m.s._ = 0.051 Å, and also reveals a mirror-image pattern of deviations (above/below plane) for sixteen of the nineteen pairs. This suggests that Ia and Ib are quasi-enantiomorphs. Indeed, a least-squares fit of the relative atomic coordinates of Ia to those of Ib inverted through the origin give an average mis-match δ_r.m.s._ = 0.022 Å.

The triclinic form of I at 123 K has been reported (Raston & Scott, 2000; CCDC refcode LOLYUG; Allen, 2002). It is also significantly non-planar with δ/σ > 3 for sixteen framework atoms. The relative atomic positions match closely those of Ia (δ_r.m.s._ = 0.051 Å) and Ib (δ_r.m.s._ = 0.050 Å), and the pattern of deviations for thirteen non-H atoms is identical to equivalent atoms in Ia.

### Experimental

The synthesis is detailed by Bell & Spanswick (1966): benzyl cyanide (1.8 g.) was added to a solution of sodium (0.35 g) in ethanol (20 ml). Indan-1-one (2 g) was then added, and the mixture warmed on a steam-bath for 20 min. The product was cooled, diluted, and acidified with acetic acid. The sticky precipitate was crystallized from ethanol to yield yellow needles, m.p. 141–143°C.

### Refinement

The absolute configuration could not be determined by analysis of 1665 Bijvoet pairs (Flack (1983) *x* = 0(2), Hooft *et al.* (2008) *y* = 0.4 (12). All H atoms were placed in calculated positions, with C—H distances of 0.95–0.99 Å, *U*_iso_ = 1.2 of the attached carbon atom, and thereafter treated as riding.

### Crystal data

**Table:**

C_18_H_14_O	*D*_x_ = 1.304 Mg m^−^^3^
*M**_r_* = 246.29	Melting point = 414–416 K
Orthorhombic, *P*2_1_2_1_2_1_	Mo *K*α radiation, λ = 0.71073 Å
Hall symbol: P 2ac 2ab	Cell parameters from 2407 reflections
*a* = 5.291 (2) Å	θ = 2.5–25°
*b* = 17.809 (5) Å	µ = 0.08 mm^−^^1^
*c* = 26.622 (9) Å	*T* = 123 K
*V* = 2508.5 (15) Å^3^	Needle, yellow
*Z* = 8	0.35 × 0.12 × 0.05 mm
*F*(000) = 1040	

### Data collection

**Table:**

Nonius KappaCCD diffractometer	4236 independent reflections
Radiation source: sealed tube	3145 reflections with *I* > 2σ(*I*)
Horizonally mounted graphite crystal monochromator	*R*_int_ = 0.074
Detector resolution: 9 pixels mm^-1^	θ_max_ = 25.0°, θ_min_ = 2.6°
ω and φ scans	*h* = −6→6
Absorption correction: multi-scan *HKL**SCALEPACK* (Otwinowski & Minor 1997)	*k* = −20→21
*T*_min_ = 0.973, *T*_max_ = 0.996	*l* = −31→31
11743 measured reflections	

### Refinement

**Table:**

Refinement on *F*^2^	Hydrogen site location: inferred from neighbouring sites
Least-squares matrix: full	H-atom parameters constrained
*R*[*F*^2^ > 2σ(*F*^2^)] = 0.057	*w* = 1/[σ^2^(*F*_o_^2^) + (0.0283*P*)^2^ + 1.1913*P*] where *P* = (*F*_o_^2^ + 2*F*_c_^2^)/3
*wR*(*F*^2^) = 0.110	(Δ/σ)_max_ = 0.001
*S* = 1.05	Δρ_max_ = 0.21 e Å^−^^3^
4236 reflections	Δρ_min_ = −0.18 e Å^−^^3^
344 parameters	Extinction correction: *SHELXL97* (Sheldrick, 2008), Fc^*^=kFc[1+0.001xFc^2^λ^3^/sin(2θ)]^-1/4^
0 restraints	Extinction coefficient: 0.0049 (8)
0 constraints	Absolute structure: Flack (1983), with 1665 Bijvoet pairs
Primary atom site location: structure-invariant direct methods	Flack parameter: 0 (2)
Secondary atom site location: difference Fourier map	

### Special details

**Table:**

Geometry. All e.s.d.'s (except the e.s.d. in the dihedral angle between two l.s. planes) are estimated using the full covariance matrix. The cell e.s.d.'s are taken into account individually in the estimation of e.s.d.'s in distances, angles and torsion angles; correlations between e.s.d.'s in cell parameters are only used when they are defined by crystal symmetry. An approximate (isotropic) treatment of cell e.s.d.'s is used for estimating e.s.d.'s involving l.s. planes.

### Fractional atomic coordinates and isotropic or equivalent isotropic displacement parameters (Å^2^)

**Table:**

	*x*	*y*	*z*	*U*_iso_*/*U*_eq_	
C1	0.3862 (6)	0.10668 (16)	0.32450 (12)	0.0256 (7)	
C2	0.5813 (5)	0.05703 (16)	0.30273 (11)	0.0244 (7)	
C3	0.7531 (6)	0.01103 (17)	0.32733 (12)	0.0300 (8)	
H3	0.7526	0.0072	0.3629	0.036*	
C4	0.9248 (6)	−0.02910 (17)	0.29896 (12)	0.0309 (8)	
H4	1.0456	−0.0603	0.3151	0.037*	
C5	0.9216 (6)	−0.02405 (17)	0.24675 (13)	0.0310 (8)	
H5	1.0391	−0.0526	0.2277	0.037*	
C6	0.7499 (6)	0.02196 (16)	0.22196 (12)	0.0269 (7)	
H6	0.7488	0.0252	0.1863	0.032*	
C7	0.5794 (6)	0.06329 (16)	0.25077 (11)	0.0239 (8)	
C8	0.3800 (5)	0.11835 (16)	0.23340 (11)	0.0238 (7)	
H8A	0.2542	0.0934	0.2115	0.029*	
H8B	0.4574	0.1607	0.2148	0.029*	
C9	0.2586 (5)	0.14540 (16)	0.28197 (11)	0.0221 (7)	
C10	0.0721 (5)	0.19601 (16)	0.28771 (11)	0.0230 (7)	
C11	−0.0697 (5)	0.23669 (15)	0.24841 (11)	0.0225 (7)	
C12	−0.0489 (6)	0.23430 (16)	0.19628 (11)	0.0257 (7)	
H12	0.0758	0.2038	0.1807	0.031*	
C13	−0.2134 (6)	0.27720 (17)	0.16749 (12)	0.0296 (8)	
H13	−0.1998	0.2763	0.1319	0.036*	
C14	−0.3968 (6)	0.32121 (17)	0.18995 (13)	0.0345 (9)	
H14	−0.5096	0.3494	0.1696	0.041*	
C15	−0.4178 (6)	0.32469 (17)	0.24150 (14)	0.0331 (8)	
H15	−0.5428	0.3555	0.2567	0.04*	
C16	−0.2536 (6)	0.28247 (16)	0.27084 (11)	0.0271 (8)	
C17	−0.2433 (6)	0.27776 (17)	0.32697 (12)	0.0313 (8)	
H17A	−0.4076	0.2609	0.3407	0.038*	
H17B	−0.2005	0.3272	0.3417	0.038*	
C18	−0.0346 (6)	0.21981 (18)	0.33823 (11)	0.0299 (8)	
H18A	0.0994	0.2425	0.3593	0.036*	
H18B	−0.1057	0.176	0.3562	0.036*	
O1	0.3417 (4)	0.11400 (12)	0.36966 (8)	0.0380 (6)	
C19	0.0040 (5)	−0.05063 (16)	0.07403 (12)	0.0248 (7)	
C20	−0.1891 (5)	−0.09719 (15)	0.04914 (11)	0.0236 (7)	
C21	−0.3649 (6)	−0.14487 (17)	0.07113 (12)	0.0293 (8)	
H21	−0.3691	−0.1517	0.1065	0.035*	
C22	−0.5337 (6)	−0.18215 (16)	0.04030 (13)	0.0343 (8)	
H22	−0.6548	−0.2153	0.0545	0.041*	
C23	−0.5264 (6)	−0.17121 (17)	−0.01129 (13)	0.0328 (8)	
H23	−0.6433	−0.1973	−0.032	0.039*	
C24	−0.3526 (6)	−0.12311 (16)	−0.03339 (12)	0.0301 (8)	
H24	−0.3498	−0.1159	−0.0687	0.036*	
C25	−0.1821 (6)	−0.08560 (16)	−0.00228 (11)	0.0237 (8)	
C26	0.0229 (5)	−0.03001 (16)	−0.01635 (11)	0.0249 (7)	
H26A	0.1504	−0.0535	−0.0386	0.03*	
H26B	−0.0494	0.0144	−0.0335	0.03*	
C27	0.1375 (5)	−0.00859 (15)	0.03373 (11)	0.0227 (7)	
C28	0.3261 (5)	0.04062 (16)	0.04303 (11)	0.0224 (7)	
C29	0.4744 (6)	0.08474 (16)	0.00679 (11)	0.0223 (7)	
C30	0.4644 (6)	0.08748 (16)	−0.04551 (11)	0.0266 (7)	
H30	0.3434	0.0585	−0.0634	0.032*	
C31	0.6341 (6)	0.13329 (17)	−0.07129 (12)	0.0307 (8)	
H31	0.6296	0.1352	−0.1069	0.037*	
C32	0.8107 (6)	0.17629 (16)	−0.04507 (13)	0.0323 (8)	
H32	0.9255	0.2072	−0.0631	0.039*	
C33	0.8205 (6)	0.17441 (17)	0.00674 (13)	0.0309 (9)	
H33	0.9406	0.204	0.0244	0.037*	
C34	0.6521 (6)	0.12866 (16)	0.03270 (11)	0.0265 (8)	
C35	0.6346 (6)	0.11740 (17)	0.08845 (11)	0.0335 (8)	
H35A	0.5896	0.1649	0.1056	0.04*	
H35B	0.797	0.0989	0.1022	0.04*	
C36	0.4246 (6)	0.05840 (17)	0.09514 (11)	0.0284 (8)	
H36A	0.4931	0.0126	0.1112	0.034*	
H36B	0.2873	0.0785	0.1165	0.034*	
O2	0.0410 (4)	−0.04698 (12)	0.11985 (8)	0.0332 (6)	

### Atomic displacement parameters (Å^2^)

**Table:**

	*U*^11^	*U*^22^	*U*^33^	*U*^12^	*U*^13^	*U*^23^
C1	0.0271 (18)	0.0229 (17)	0.027 (2)	−0.0017 (15)	−0.0014 (14)	0.0003 (14)
C2	0.0230 (17)	0.0179 (16)	0.032 (2)	−0.0054 (15)	−0.0018 (14)	0.0011 (14)
C3	0.0305 (18)	0.0240 (18)	0.035 (2)	0.0015 (16)	−0.0022 (16)	0.0078 (15)
C4	0.0235 (17)	0.0214 (17)	0.048 (2)	0.0034 (16)	−0.0030 (16)	0.0088 (15)
C5	0.0241 (18)	0.0195 (17)	0.049 (2)	0.0000 (16)	0.0031 (16)	0.0010 (15)
C6	0.0246 (16)	0.0242 (17)	0.0318 (19)	−0.0042 (15)	0.0016 (15)	−0.0020 (15)
C7	0.0224 (17)	0.0158 (16)	0.033 (2)	−0.0026 (15)	0.0001 (15)	0.0020 (14)
C8	0.0238 (16)	0.0206 (16)	0.0269 (17)	−0.0014 (14)	0.0028 (13)	−0.0004 (13)
C9	0.0232 (16)	0.0210 (17)	0.0221 (18)	−0.0033 (15)	0.0001 (14)	−0.0006 (13)
C10	0.0237 (16)	0.0164 (16)	0.0290 (19)	−0.0032 (14)	0.0016 (14)	−0.0019 (13)
C11	0.0188 (17)	0.0147 (15)	0.034 (2)	−0.0014 (14)	−0.0013 (14)	−0.0006 (14)
C12	0.0248 (17)	0.0223 (16)	0.030 (2)	−0.0030 (15)	−0.0027 (14)	0.0000 (14)
C13	0.0284 (19)	0.0222 (17)	0.038 (2)	−0.0068 (16)	−0.0041 (15)	0.0059 (15)
C14	0.030 (2)	0.0236 (18)	0.050 (2)	−0.0043 (17)	−0.0148 (17)	0.0068 (16)
C15	0.0239 (18)	0.0188 (17)	0.057 (3)	0.0040 (15)	−0.0060 (17)	−0.0035 (16)
C16	0.0216 (16)	0.0212 (17)	0.039 (2)	−0.0073 (15)	−0.0018 (15)	−0.0019 (15)
C17	0.0240 (18)	0.0239 (18)	0.046 (2)	0.0019 (15)	0.0042 (16)	−0.0073 (15)
C18	0.0306 (18)	0.0306 (18)	0.0286 (19)	0.0003 (16)	0.0035 (14)	−0.0065 (14)
O1	0.0468 (15)	0.0413 (14)	0.0259 (14)	0.0089 (12)	0.0002 (11)	0.0016 (11)
C19	0.0214 (17)	0.0203 (17)	0.033 (2)	0.0077 (14)	−0.0001 (15)	0.0031 (14)
C20	0.0206 (17)	0.0151 (16)	0.035 (2)	0.0042 (14)	0.0008 (14)	0.0011 (14)
C21	0.0242 (17)	0.0272 (18)	0.037 (2)	0.0025 (16)	0.0068 (15)	0.0077 (15)
C22	0.0290 (19)	0.0192 (17)	0.055 (2)	−0.0037 (15)	0.0060 (17)	0.0014 (16)
C23	0.0254 (19)	0.0219 (18)	0.051 (2)	−0.0032 (16)	−0.0032 (17)	−0.0083 (16)
C24	0.033 (2)	0.0221 (17)	0.035 (2)	0.0000 (17)	−0.0015 (15)	−0.0077 (15)
C25	0.0198 (18)	0.0173 (16)	0.034 (2)	0.0037 (14)	0.0020 (13)	0.0000 (14)
C26	0.0261 (17)	0.0217 (16)	0.0268 (18)	0.0027 (15)	0.0031 (14)	−0.0007 (14)
C27	0.0230 (17)	0.0183 (16)	0.0267 (19)	0.0041 (15)	0.0018 (13)	−0.0023 (13)
C28	0.0182 (16)	0.0205 (16)	0.0286 (18)	0.0074 (15)	0.0004 (13)	−0.0011 (14)
C29	0.0178 (17)	0.0165 (16)	0.033 (2)	0.0044 (14)	0.0011 (14)	−0.0009 (14)
C30	0.0228 (17)	0.0242 (17)	0.033 (2)	0.0012 (15)	−0.0012 (14)	0.0008 (14)
C31	0.0285 (18)	0.0272 (18)	0.036 (2)	0.0068 (16)	0.0029 (15)	0.0061 (15)
C32	0.0263 (19)	0.0189 (17)	0.052 (2)	0.0001 (15)	0.0062 (16)	0.0080 (15)
C33	0.029 (2)	0.0200 (18)	0.044 (2)	0.0003 (16)	0.0005 (16)	−0.0002 (15)
C34	0.0280 (18)	0.0170 (16)	0.035 (2)	0.0039 (16)	−0.0004 (14)	−0.0005 (14)
C35	0.0361 (19)	0.0253 (18)	0.039 (2)	−0.0027 (17)	−0.0050 (16)	0.0001 (15)
C36	0.0246 (18)	0.0306 (19)	0.0300 (19)	−0.0039 (15)	0.0002 (15)	−0.0012 (14)
O2	0.0327 (13)	0.0400 (14)	0.0268 (14)	−0.0005 (11)	0.0007 (10)	0.0030 (10)

### Geometric parameters (Å, º)

**Table:**

C1—O1	1.232 (3)	C19—O2	1.237 (3)
C1—C2	1.478 (4)	C19—C20	1.473 (4)
C1—C9	1.488 (4)	C19—C27	1.487 (4)
C2—C3	1.388 (4)	C20—C25	1.385 (4)
C2—C7	1.388 (4)	C20—C21	1.389 (4)
C3—C4	1.381 (4)	C21—C22	1.383 (4)
C3—H3	0.95	C21—H21	0.95
C4—C5	1.393 (4)	C22—C23	1.388 (4)
C4—H4	0.95	C22—H22	0.95
C5—C6	1.390 (4)	C23—C24	1.388 (4)
C5—H5	0.95	C23—H23	0.95
C6—C7	1.394 (4)	C24—C25	1.395 (4)
C6—H6	0.95	C24—H24	0.95
C7—C8	1.513 (4)	C25—C26	1.516 (4)
C8—C9	1.522 (4)	C26—C27	1.514 (4)
C8—H8A	0.99	C26—H26A	0.99
C8—H8B	0.99	C26—H26B	0.99
C9—C10	1.345 (4)	C27—C28	1.351 (4)
C10—C11	1.477 (4)	C28—C29	1.471 (4)
C10—C18	1.519 (4)	C28—C36	1.515 (4)
C11—C12	1.393 (4)	C29—C30	1.394 (4)
C11—C16	1.403 (4)	C29—C34	1.404 (4)
C12—C13	1.389 (4)	C30—C31	1.394 (4)
C12—H12	0.95	C30—H30	0.95
C13—C14	1.383 (4)	C31—C32	1.395 (4)
C13—H13	0.95	C31—H31	0.95
C14—C15	1.378 (5)	C32—C33	1.381 (4)
C14—H14	0.95	C32—H32	0.95
C15—C16	1.389 (4)	C33—C34	1.391 (4)
C15—H15	0.95	C33—H33	0.95
C16—C17	1.498 (4)	C34—C35	1.501 (4)
C17—C18	1.541 (4)	C35—C36	1.540 (4)
C17—H17A	0.99	C35—H35A	0.99
C17—H17B	0.99	C35—H35B	0.99
C18—H18A	0.99	C36—H36A	0.99
C18—H18B	0.99	C36—H36B	0.99
			
O1—C1—C2	125.4 (3)	O2—C19—C20	125.7 (3)
O1—C1—C9	127.4 (3)	O2—C19—C27	127.6 (3)
C2—C1—C9	107.2 (2)	C20—C19—C27	106.8 (3)
C3—C2—C7	121.5 (3)	C25—C20—C21	121.7 (3)
C3—C2—C1	128.7 (3)	C25—C20—C19	110.0 (3)
C7—C2—C1	109.7 (3)	C21—C20—C19	128.2 (3)
C4—C3—C2	118.6 (3)	C22—C21—C20	118.4 (3)
C4—C3—H3	120.7	C22—C21—H21	120.8
C2—C3—H3	120.7	C20—C21—H21	120.8
C3—C4—C5	120.3 (3)	C21—C22—C23	120.2 (3)
C3—C4—H4	119.9	C21—C22—H22	119.9
C5—C4—H4	119.9	C23—C22—H22	119.9
C6—C5—C4	121.3 (3)	C22—C23—C24	121.6 (3)
C6—C5—H5	119.3	C22—C23—H23	119.2
C4—C5—H5	119.3	C24—C23—H23	119.2
C5—C6—C7	118.2 (3)	C23—C24—C25	118.2 (3)
C5—C6—H6	120.9	C23—C24—H24	120.9
C7—C6—H6	120.9	C25—C24—H24	120.9
C2—C7—C6	120.1 (3)	C20—C25—C24	119.9 (3)
C2—C7—C8	111.2 (3)	C20—C25—C26	111.1 (3)
C6—C7—C8	128.7 (3)	C24—C25—C26	129.0 (3)
C7—C8—C9	103.9 (2)	C27—C26—C25	103.5 (2)
C7—C8—H8A	111	C27—C26—H26A	111.1
C9—C8—H8A	111	C25—C26—H26A	111.1
C7—C8—H8B	111	C27—C26—H26B	111.1
C9—C8—H8B	111	C25—C26—H26B	111.1
H8A—C8—H8B	109	H26A—C26—H26B	109
C10—C9—C1	123.9 (3)	C28—C27—C19	123.0 (3)
C10—C9—C8	128.2 (3)	C28—C27—C26	128.4 (3)
C1—C9—C8	107.9 (2)	C19—C27—C26	108.6 (2)
C9—C10—C11	128.4 (3)	C27—C28—C29	128.3 (3)
C9—C10—C18	124.1 (3)	C27—C28—C36	123.9 (3)
C11—C10—C18	107.6 (2)	C29—C28—C36	107.8 (2)
C12—C11—C16	119.8 (3)	C30—C29—C34	119.8 (3)
C12—C11—C10	130.6 (3)	C30—C29—C28	130.8 (3)
C16—C11—C10	109.6 (3)	C34—C29—C28	109.4 (3)
C13—C12—C11	118.9 (3)	C31—C30—C29	119.2 (3)
C13—C12—H12	120.5	C31—C30—H30	120.4
C11—C12—H12	120.5	C29—C30—H30	120.4
C14—C13—C12	120.8 (3)	C30—C31—C32	120.4 (3)
C14—C13—H13	119.6	C30—C31—H31	119.8
C12—C13—H13	119.6	C32—C31—H31	119.8
C15—C14—C13	120.8 (3)	C33—C32—C31	120.8 (3)
C15—C14—H14	119.6	C33—C32—H32	119.6
C13—C14—H14	119.6	C31—C32—H32	119.6
C14—C15—C16	119.0 (3)	C32—C33—C34	119.1 (3)
C14—C15—H15	120.5	C32—C33—H33	120.5
C16—C15—H15	120.5	C34—C33—H33	120.5
C15—C16—C11	120.6 (3)	C33—C34—C29	120.7 (3)
C15—C16—C17	127.9 (3)	C33—C34—C35	127.5 (3)
C11—C16—C17	111.5 (3)	C29—C34—C35	111.7 (3)
C16—C17—C18	104.9 (2)	C34—C35—C36	104.5 (2)
C16—C17—H17A	110.8	C34—C35—H35A	110.9
C18—C17—H17A	110.8	C36—C35—H35A	110.9
C16—C17—H17B	110.8	C34—C35—H35B	110.9
C18—C17—H17B	110.8	C36—C35—H35B	110.9
H17A—C17—H17B	108.8	H35A—C35—H35B	108.9
C10—C18—C17	106.3 (2)	C28—C36—C35	106.6 (2)
C10—C18—H18A	110.5	C28—C36—H36A	110.4
C17—C18—H18A	110.5	C35—C36—H36A	110.4
C10—C18—H18B	110.5	C28—C36—H36B	110.4
C17—C18—H18B	110.5	C35—C36—H36B	110.4
H18A—C18—H18B	108.7	H36A—C36—H36B	108.6
			
O1—C1—C2—C3	−1.5 (5)	O2—C19—C20—C25	178.1 (3)
C9—C1—C2—C3	178.2 (3)	C27—C19—C20—C25	−0.6 (3)
O1—C1—C2—C7	−179.7 (3)	O2—C19—C20—C21	0.3 (5)
C9—C1—C2—C7	0.0 (3)	C27—C19—C20—C21	−178.3 (3)
C7—C2—C3—C4	0.0 (5)	C25—C20—C21—C22	1.0 (4)
C1—C2—C3—C4	−178.0 (3)	C19—C20—C21—C22	178.5 (3)
C2—C3—C4—C5	−1.0 (5)	C20—C21—C22—C23	−0.5 (4)
C3—C4—C5—C6	1.0 (5)	C21—C22—C23—C24	−0.1 (5)
C4—C5—C6—C7	0.0 (4)	C22—C23—C24—C25	0.2 (5)
C3—C2—C7—C6	1.0 (4)	C21—C20—C25—C24	−0.9 (4)
C1—C2—C7—C6	179.4 (2)	C19—C20—C25—C24	−178.8 (2)
C3—C2—C7—C8	−178.4 (3)	C21—C20—C25—C26	178.8 (3)
C1—C2—C7—C8	0.0 (3)	C19—C20—C25—C26	0.8 (3)
C5—C6—C7—C2	−1.0 (4)	C23—C24—C25—C20	0.3 (4)
C5—C6—C7—C8	178.3 (3)	C23—C24—C25—C26	−179.3 (3)
C2—C7—C8—C9	0.0 (3)	C20—C25—C26—C27	−0.7 (3)
C6—C7—C8—C9	−179.3 (3)	C24—C25—C26—C27	178.9 (3)
O1—C1—C9—C10	0.7 (5)	O2—C19—C27—C28	0.6 (5)
C2—C1—C9—C10	−179.0 (3)	C20—C19—C27—C28	179.2 (3)
O1—C1—C9—C8	179.7 (3)	O2—C19—C27—C26	−178.5 (3)
C2—C1—C9—C8	0.0 (3)	C20—C19—C27—C26	0.1 (3)
C7—C8—C9—C10	178.9 (3)	C25—C26—C27—C28	−178.7 (3)
C7—C8—C9—C1	0.0 (3)	C25—C26—C27—C19	0.4 (3)
C1—C9—C10—C11	−179.0 (3)	C19—C27—C28—C29	179.2 (3)
C8—C9—C10—C11	2.3 (5)	C26—C27—C28—C29	−1.9 (5)
C1—C9—C10—C18	−0.6 (4)	C19—C27—C28—C36	0.4 (4)
C8—C9—C10—C18	−179.4 (3)	C26—C27—C28—C36	179.4 (3)
C9—C10—C11—C12	−0.5 (5)	C27—C28—C29—C30	−0.8 (5)
C18—C10—C11—C12	−179.0 (3)	C36—C28—C29—C30	178.1 (3)
C9—C10—C11—C16	178.1 (3)	C27—C28—C29—C34	−179.8 (3)
C18—C10—C11—C16	−0.4 (3)	C36—C28—C29—C34	−0.9 (3)
C16—C11—C12—C13	−0.5 (4)	C34—C29—C30—C31	0.8 (4)
C10—C11—C12—C13	178.0 (3)	C28—C29—C30—C31	−178.1 (3)
C11—C12—C13—C14	−0.5 (4)	C29—C30—C31—C32	−0.5 (4)
C12—C13—C14—C15	1.2 (5)	C30—C31—C32—C33	−0.1 (5)
C13—C14—C15—C16	−0.8 (5)	C31—C32—C33—C34	0.3 (5)
C14—C15—C16—C11	−0.3 (5)	C32—C33—C34—C29	0.1 (4)
C14—C15—C16—C17	−179.9 (3)	C32—C33—C34—C35	178.7 (3)
C12—C11—C16—C15	0.9 (4)	C30—C29—C34—C33	−0.7 (4)
C10—C11—C16—C15	−177.9 (3)	C28—C29—C34—C33	178.5 (3)
C12—C11—C16—C17	−179.4 (3)	C30—C29—C34—C35	−179.5 (3)
C10—C11—C16—C17	1.8 (3)	C28—C29—C34—C35	−0.3 (3)
C15—C16—C17—C18	177.2 (3)	C33—C34—C35—C36	−177.3 (3)
C11—C16—C17—C18	−2.4 (3)	C29—C34—C35—C36	1.4 (3)
C9—C10—C18—C17	−179.7 (3)	C27—C28—C36—C35	−179.3 (3)
C11—C10—C18—C17	−1.0 (3)	C29—C28—C36—C35	1.7 (3)
C16—C17—C18—C10	2.0 (3)	C34—C35—C36—C28	−1.8 (3)
